# Association between childhood sexual abuse and early sexual debut among Chinese adolescents: The role of sexual and reproductive health education

**DOI:** 10.3389/frph.2022.909128

**Published:** 2023-01-23

**Authors:** Xu Wen, Ruoxi Ding, Chao Guo, Xiaoying Zheng

**Affiliations:** ^1^ College of Biochemical Engineering, Beijing Union University, Beijing, China; ^2^ China Center for Health Development Studies, Peking University, Beijing, China; ^3^ APEC Health Science Academy, Peking University, Beijing, China

**Keywords:** childhood sexual abuse (CSA), early sexual debut, sexual and reproductive health education, China, nationally representative survey

## Abstract

**Background:**

Despite accumulating evidence of the long-term impacts of childhood sexual abuse (CSA), few studies have investigated the association between CSA and early sexual debut among adolescents. In this study, we examine the relationship between CSA and early initiation of sexual intercourse among Chinese youth, and the role of school-based sex education in this association, based on a nationally representative survey.

**Methods:**

Data were collected from the Survey of Youth Access to Reproductive Health in China (YARHC) conducted in 2009. Multivariable logistic regression models were used to investigate the association between CSA experience and early sexual debut, and the interaction terms between sexual and reproductive health education and CSA were included to examine the role of education in the association between CSA and early sexual debut.

**Results:**

Among 4,907 sexually experienced youth, 1,062 (21.6%) made their early sexual debut. After adjusting for sociodemographic characteristics, it was found that CSA experience was significantly associated with early sexual debut, with an adjusted odds ratio of 3.13 (95% CI: 1.67–5.87). Receiving any type of sexuality education (reproductive health, sexually transmitted disease (STD) and HIV prevention, or contraception use) was not associated with a decreased risk of early sexual debut.

**Conclusion:**

Our results indicate a greater risk of early sexual debut among Chinese adolescents with a history of CSA, and only 46.7% sexually experienced youth had received prior sex education, which suggested an inadequacy of school-based sexuality education. To reduce this risk, targeted intervention with timely and adequate sexuality education for both early starters of sexual intercourse and CSA victims is warranted in China.

## Introduction

The development of adolescent sexuality is of great concern to families and society. As a major milestone in life course, the sexual debut of adolescents has been considered as a basis for their subsequent patterns of sexual psychology and behavior. The age of sexual debut among adolescents and youth around the world saw a decline in the past few decades (1, 2). A substantial proportion of adolescents initiated sexual intercourse at an age without reaching a certain level of maturity and cognitive ability to properly handle the circumstance (3). Early sexual debut has been demonstrated to be associated with various sexual risk factors such as multiple sexual partners, reduced use of contraception, and sexual intercourse after alcohol use, which are further linked to subsequent negative outcomes such as sexual transmitted diseases, unintended pregnancy, and even suicide attempts (4–6). Moreover, it has been suggested as an important predictor for undesirable social consequences in later life. Individuals who initiate sexual intercourse in early adolescence are more likely to be in a condition of poor health, compromised economic conditions, and reduced life satisfaction (7).

As one of the major types of adverse childhood experiences, sexual abuse during childhood has been suggested to be associated with multiple adverse health risk behaviors and outcomes such as risky sexual behaviors (8), teenage pregnancy (9), and problems connected with intimacy relationships (10), to name a few. A previous study has indicated that sexual trauma may disrupt the psychosocial development of abused children, operate through cognitive and emotional processes, and further manifest in behaviors (11, 12). Some amount of research seems to suggest that early sexual debut plays a mediating role in the association between childhood sexual abuse (CSA) and risky sexual behaviors in later life (13). However, despite the accumulating evidence of the long-term impacts of sexual trauma, few studies have investigated the association between CSA and early sexual debut among adolescents. Considering the significant influence of sexual debut on individuals’ life-time sexual trajectory, evidence regarding the effect of childhood sexual abuse on the development of sexual activity is absolutely essential for making targeted interventions.

It is widely recognized that sex education is an essential component of healthy physical and psychosocial development of adolescents and young adults (14). A study from the United States has shown that the implementation of sex education can reduce the risk of early initiation of sexual activity among adolescents (15). Comprehensive education of sexual health not only provides the knowledge and skills necessary to help young people make informed choices of sexual and reproductive behaviors and offers useful linkage and resources to clinical services, but also helps adolescents to form healthy relationships and set goals and plans during their transition from adolescence to adulthood (16). Meanwhile, sex education is also recommended as a key component in the treatment of CSA victims (17). An analysis of the CSA intervention programs suggests that sexual and reproductive health education (SRHE) is necessary for reducing negative emotions related to abuse experience and promoting healthy intimate relationships for the victims (17). Thus, the impact of sex education on the timing of sexual debut among CSA victims is essential for the formulation and adjustment of treatment programs. However, little research has been conducted on providing empirical evaluation.

Marked changes have occurred in people's attitudes toward sex and sexual expression in China since the launch of the reform and opening-up policy (18). A study of Chinese university students in 2005 showed that the peak age of sexual debut was 17–19 years, which significantly declined compared with 20–22 years of age a decade ago (19, 20). In the meantime, childhood sexual abuse was not and is not an unusual phenomenon in China. According to an estimation in a systematic review, in the 1988–2013 period, 8.7% of Chinese children under the age of 18 years suffered from sexual abuse (21). Nevertheless, it has been pointed out that the implementation of sex education in China was insufficient in the past and has also failed to keep pace with the physical and social changes that have taken place over the years, leaving the new generation of youth at risk of adverse sexual consequences (22). Formal sexuality education in Chinese schools is widely considered to be inadequate because of its insufficient class hours and a lack of teacher resources (23), which is evident from the fact that 44.4% of Chinese college students reported having never received prior school-based sexuality education (24).

Overall, nearly half of the respondents in this survey reported having never received school-based sexuality education. For the other half, sexuality education was mainly conducted during middle school and college years.

Therefore, in this study, we examined the relationship between CSA and early initiation of sexual intercourse among Chinese youth, and the role of school-based sex education in this association, based on a nationally representative survey.

## Methods

### Data source and ethical approval

Data were collected from the Survey of Youth Access to Reproductive Health in China (YARHC). This nationally representative and population-based survey was conducted by Peking University in 2009. It aimed to describe the level of knowledge on, and the attitude and behavior toward, sexual and reproductive health among unmarried youth in mainland China and to explore their accessibility to reproductive health services. Experts from related academic fields in China and the United Nations Fund for Population Activities (UNFPA) designed the study protocol and questionnaire of this survey. The Institutional Review Board of Peking University Health Science Center reviewed and approved this study (No. 20090928). Consent to participate was obtained from all respondents, and for those under 18 years, the consent form was signed by their adult guardians.

### Participants and samples

In the YARHC survey, the target population was unmarried youth aged 15–24 years living in mainland China. Three subpopulations were included: school youth (in school, either living on campus or in a community), household youth (living with family, either employed or unemployed), and collective household youth (employed, living in a communal house) to cover participants from different environments. All interviewers received rigorous training by a committee of survey experts, who used the standard set, and a pilot study with preliminary interviews was conducted before the formal survey. The privacy of youth was protected during the entire process of the survey, with all participants interviewed face-to-face in independent environments without the presence of a third party. For the section on sensitive topics related to sexual experiences and behaviors, the questionnaire was self-administered and completed by individuals themselves. In addition, the respondents were interviewed by those of the same sex.

**Figure 1:**
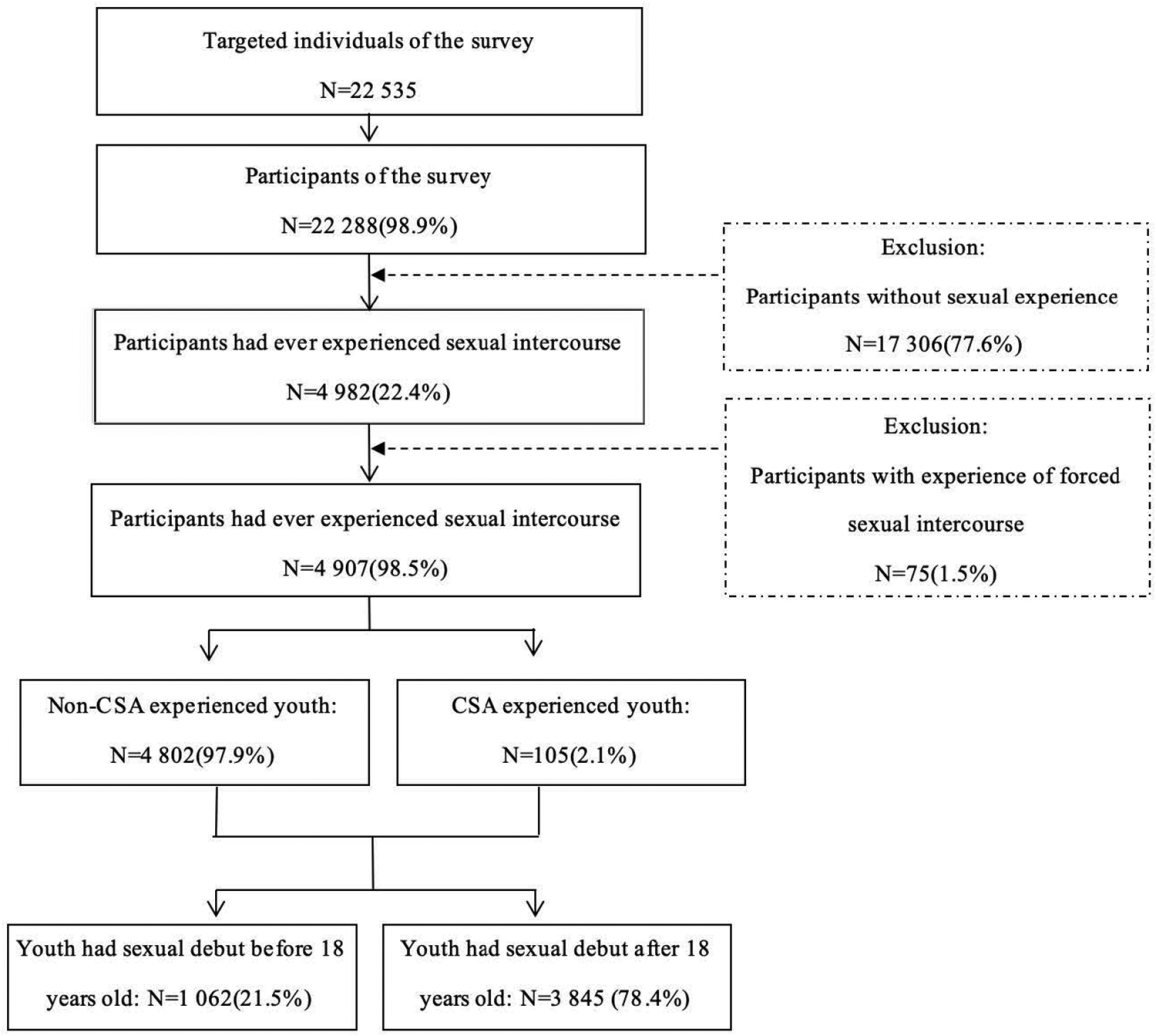
Flowchart of the study sample.

This survey applied a four-stage stratified random cluster sampling with probability proportional to size. The four stages for school youth, household youth, and collective household youth were cities-schools-classes-students, cities-counties-communities-household youth, and cities-counties-map pieces-collective household youth, respectively. The final estimated sample size was distributed in 40 cities and counties from 25 provinces/autonomous regions/municipalities in China. When the selected subject could not be contacted, or there was no target youth in the selected household, the interviewers followed the sample substitution principle, in which the neighbor of the nonrespondents would be taken as a replacement, with a swing priority from right to left within five units. Given the refusal rate of 24.9%, 22,465 questionnaires were finally collected, of which 22,288 were valid, achieving a validity rate of 98.9%. Because we focused on the association between CSA and early initiation of sexual intercourse, our study sample was restricted to subjects who were asked whether they have ever had sexual intercourse and never experienced forced sexual intercourse (to avoid a case where the sexual debut was forced sexual intercourse), and a total of 4,907 cases were included in the analysis (Figure 1).

#### Patient and public involvement

Patients or members of the public *were not* involved in the design, conduct, reporting, or dissemination plans of our research.

### Measures

#### Related sexual experience

The question “Have you ever had a sexual intercourse (whether it was with your current/latest girlfriend/boyfriend or with any others)?” was employed to determine whether a respondent was sexually experienced; if the answer was “yes,” then the participant was rated as sexually experienced. Also, the question “Some young people are forced to have sexual intercourse with others (non-boy/girlfriends). Have you ever had such an experience?” was employed to determine whether an individual had ever had forced sexual intercourse. The above two variables were used to exclude respondents without sexual experience or respondents who experienced forced sexual intercourse.

#### Childhood sexual abuse experience (independent variable)

In the YARHC survey, the question “Some young people are forced to touch, or be touched on, the sensitive body parts by others (non-boy/girlfriends). Have you ever had such an experience?” was employed to determine whether an individual had ever been sexually abused. If the answer was “yes,” the age when the first sexual abuse happened was extracted from the question “How old were you when these things first happened?” When the age was younger than 18 years, the respondent was defined as having experienced CSA.

#### Early sexual debut (dependent variable)

The question “How old were you when you had sexual intercourse for the very first time?” was employed to determine the age of sexual debut. When the age was younger than 18 years, the respondent was defined as having early sexual debut.

#### Sexual and reproductive health education

Individuals’ experience of SRHE was measured in terms of three aspects: *reproductive health education* (Have you ever attended the lecture on reproductive health education in school?), *STD and HIV prevention education* (Have you ever attended the lecture on STD and HIV prevention education in school?), and *contraception use education* (Have you ever attended the lecture on contraception use education in school?). We also used “any SRHE” to include any of the above three aspects. All four variables were binary (with yes or no answers).

#### Sociodemographic information (control variables)

Age at the time of survey was set as a continuous variable. Survey youth were categorized by sex (male or female), residence (rural or urban areas), region (east, central, or west), education level (junior high school and below, senior high school, or college and above), employment status (student, unemployed and not at school, or employed), mother's education level (junior high school and below, senior high school, or college and above), and annual family income tertiles (low, middle, or high).

### Statistical analysis

Descriptive statistics with chi-square test were applied to present the sample characteristics, population numbers, prevalence, and proportions and to examine the difference between groups where appropriate. Multivariable logistic regression models were used to calculate the adjusted odds ratios (ORs) and 95% confidence intervals (CIs) for determining the association between CSA experience and early sexual debut in Model 1. In Model 2–5, the interaction terms between each type of SRHE and CSA were included in the multivariable logistic regression models to examine the role of education in the association between CSA and early sexual debut. STATA 14 (STATA Corp, College Station, TX, United States) was used to perform all data analyses. A *P*-value of less than 0.05 was considered statistically significant.

## Results

### Sample characteristics and CSA experience among Chinese unmarried youth

Among 4,907 sexually experienced youth, 2,835 (57.8%) were male, and the mean age was 20.8 years. There were 2,008 (40.9%) from rural areas and 1,838 (37.5%) youth with a college and above degree. A total of 105 youth (2.1%) experienced CSA, of which 44 (1.5%) were male and 61 (2.9%) were female. Other demographic and socioeconomic characteristics of the study sample in terms of whole samples, male subsamples, and female subsamples are listed in Table 1.

**Table 1:** Characteristics of the study sample by gender.

	Total *N* = 4,907	Male *N* = 2,835	Female *N* = 2,072
Age, years, mean (SD)	20.8 (2.3)	20.8 (2.3)	20.9 (2.3)
Residence, *N* (%)			
Rural	2,008 (40.9)	1,164 (41.1)	844 (40.7)
Urban	2,899 (59.1)	1,671 (58.9)	1,228 (59.3)
Region, *N* (%)			
East	2,549 (51.9)	1,456 (51.4)	1,093 (52.8)
Central	1,235 (25.2)	718 (25.3)	517 (24.9)
West	1,123 (22.9)	611 (23.3)	462 (22.3)
Education, *N* (%)			
Junior high school and below	742 (15.1)	425 (15.0)	317 (15.3)
Senior high school	2,327 (47.4)	1,334 (47.0)	993 (47.9)
College and above	1,838 (37.5)	1,076 (38.0)	762 (36.8)
Employment, *N* (%)			
Student	1,564 (31.9)	975 (34.4)	589 (28.4)
Employed	2,751 (56.1)	1,541 (54.4)	1,210 (58.4)
Unemployed	592 (12.0)	319 (11.2)	273 (13.2)
Family income tertiles, *N* (%)			
Low	1,389 (28.3)	777 (27.4)	612 (29.5)
Middle	1,863 (38.0)	1,093 (38.6)	770 (37.2)
High	1,655 (33.7)	965 (34.0)	690 (33.3)
Mother's education, *N* (%)			
Junior high school and below	3,091 (63.0)	1,792 (63.2)	1,299 (62.7)
Senior high school	1,320 (26.9)	750 (26.9)	570 (27.5)
College and above	496 (10.1)	293 (10.3)	203 (9.8)
CSA experience, *N* (%)			
No	4,802 (97.9)	2,791 (98.5)	2,011 (97.1)
Yes	105 (2.1)	44 (1.5)	61 (2.9)

CSA, childhood sexual abuse.

### SRHE of Chinese youth by CSA experience

Among 4,907 sexually experienced youth, only 2,289 (46.7%) received any type of SRHE in school, 2,039 (41.5%) received reproductive health education, 786 (16.0%) received STD and HIV prevention education, and 328 (6.7%) received contraception use education in school.

The proportion of youth who received any type of SRHE, reproductive health education, STD and HIV prevention education, and contraception use education among CSA-experienced youth were 61.9%, 50.5%, 30.5%, and 12.4%, respectively, which were higher than those (46.3%, 41.4%, 15.7%, and 6.6%, respectively) among youth without CSA experience. Except reproductive health education (*P* = 0.061), chi-square tests showed significant differences in the proportion of any type of SRHE (*P* = 0.002), STD and HIV prevention (*P* < 0.001), and contraception use education (*P* = 0.018) among youth with or without CSA experience (Table 2).

**Table 2:** SRHE of Chinese youth by CSA experience.

SRHE attainment	Total, *N* = 4,907	Non-CSA-experienced youth *N* = 4,802	CSA-experienced youth, *N* = 105	*P*-value
Any SRHE, *N* (%)				0.002
No	2,618 (53.4)	2,578 (53.7)	40 (38.1)	
Yes	2,289 (46.7)	2,224 (46.3)	65 (61.9)	
Reproductive health, *N* (%)				0.061
No	2,868 (58.5)	2,816 (58.6)	52 (49.5)	
Yes	2,039 (41.5)	1,986 (41.4)	53 (50.5)	
STD and HIV prevention, *N* (%)				<0.001
No	4,121 (84.0)	4,048 (84.3)	73 (69.5)	
Yes	786 (16.0)	754 (15.7)	32 (30.5)	
Contraception use, *N* (%)				0.018
No	4,579 (93.3)	4,487 (93.4)	92 (87.6)	
Yes	328 (6.7)	315 (6.6)	13 (12.4)	

SRHE, sexual and reproductive health education; CSA, childhood sexual abuse.

### Prevalence of early sexual debut by CSA experience and SRHE among Chinese youth

**Figure 2:**
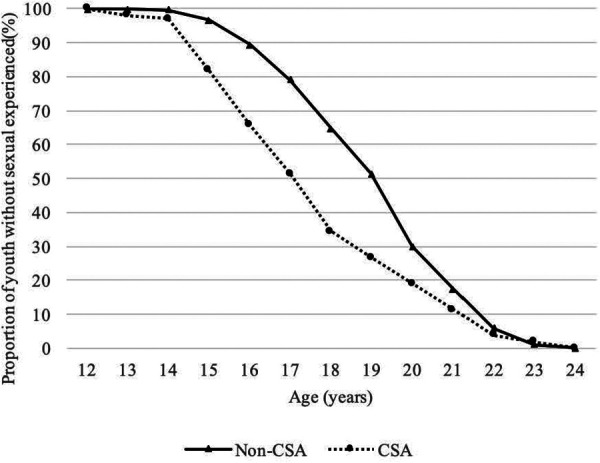
Proportions of Chinese youth who have never had a sexual experience by CSA exposure and age. CSA, childhood sexual abuse.

Among 4,907 sexually experienced youth, 1,062 (21.6%) made their early sexual debut. Among 105 CSA-experienced youth, 48.6% had early sexual debut, which was significantly higher than that (21.1%) among youth without CSA experience (*P* < 0.001). The timing of sexual debut tended to be earlier for CSA-experienced youth than for those without CSA experience (Figure 2).

As displayed in Table 3, the prevalence of early sexual debut was 23.1% among youth who received any type of SRHE in school, which was significantly higher than that (20.3%) among those without any SRHE attainment (*P* = 0.016). Similar patterns were observed for STD and HIV prevention education and contraception use education.

**Table 3:** Prevalence of early sexual debut by CSA experience and SRHE.

SRHE attainment	Total		CSA-experienced youth	
	Non-early sexual debut, *N* = 3,845	Early sexual debut, *N* = 1,062	Non-early sexual debut, *N* = 54	Early sexual debut, *N* = 51
Any SRHE, *N* (%)				
No	2,086 (79.7)	532 (20.3)	20 (50.0)	20 (50.0)
Yes	1,759 (76.9)	550 (23.1)	34 (52.3)	31 (47.7)
*P*-value	0.016		0.818	
Reproductive health				
No	2,260 (78.8)	608 (21.2)	23 (44.2)	29 (55.8)
Yes	1,585 (77.7)	454 (22.3)	31 (58.5)	22 (41.5)
*P*-value	0.371		0.144	
STD and HIV prevention				
No	3,272 (79.4)	849 (20.6)	39 (53.4)	34 (46.6)
Yes	573 (72.9)	213 (27.1)	15 (46.9)	17 (53.1)
*P*-value	<0.001		0.537	
Contraception use				
No	3,627 (79.2)	952 (20.8)	51 (55.4)	41 (44.6)
Yes	218 (66.5)	110 (33.5)	3 (23.1)	10 (76.9)
* P*-value	<0.001		0.029	

SRHE, sexual and reproductive health education; CSA, childhood sexual abuse.

For CSA-experienced youth, the prevalence of early sexual debut was 47.7%, 41.5%, 53.1%, and 76.9% among youth who received any type of SRHE, reproductive health education, STD and HIV prevention education, and contraception use education in school, respectively. Also, there was a significant difference in the prevalence of early sexual debut between youth who received contraception use education and those who did not (*P* = 0.029).

### Association between CSA, SRHE, and early sexual debut among Chinese youth

Table 4 presents the results of multivariate regression analysis for determining the association between CSA experience and early initiation of sexual intercourse. In Model 1, after adjusting for sociodemographic characteristics, it was found that CSA experience was significantly associated with early sexual debut, with an adjusted odds ratio of 3.13 (95% CI: 1.67–5.87). The risk of having an early sexual debut was higher among those who received STD and HIV prevention education (aOR: 1.49, 95% CI: 1.08–2.05) and contraception use education (aOR: 1.60, 95% CI: 1.06–2.42) than that among their counterparts without such education attainment. Model 2–Model 5 included the interaction term between CSA and each type of SRHE in the logistic regression. No significant association in the interaction term was observed in all models.

**Table 4:** Association between CSA experience, sexual and SRHE, and early sexual debut: adjusted odds ratio and 95% CI.

Variables	Age of sexual debut < 18, odds ratio (95% CI)				
	Model 1	Model 2	Model 3	Model 4	Model 5
CSA experience					
No	1	1	1	1	1
Yes	3.13 (1.67–5.87)***	5.39 (2.17–13.39)***	4.53 (1.95–10.52)***	4.50 (2.15–9.45)***	3.11 (1.60–6.05)***
Any SRHE					
No	1	1	1	1	1
Yes	0.75 (0.45–1.23)	0.76 (0.46–1.26)	0.74 (0.45–1.22)	0.74 (0.45–1.21)	0.75 (0.45–1.23)
Reproductive health					
No	1	1	1	1	1
Yes	0.95 (0.60–1.51)	0.95 (0.60–1.51)	0.98 (0.62–1.56)	0.97 (0.61–1.53)	0.95 (0.60–1.51)
STD and HIV prevention					
No	1	1	1	1	1
Yes	1.49 (1.08–2.05)***	1.50 (1.09–2.07)*	1.51 (1.09–2.08)*	1.56 (1.13–2.16)**	1.49 (1.08–2.05)*
Contraception use					
No	1	1	1	1	1
Yes	1.60 (1.06–2.42)***	1.61 (1.06–2.43)*	1.59 (1.05–2.40)*	1.60 (1.06–2.41)*	1.60 (1.05–2.43)*
CSA*: any SRHE					
Yes*	—	0.36 (0.10–1.24)	—	—	—
CSA*: reproductive health					
Yes*	—	—	0.43 (0.12–1.53)	—	—
CSA*: STD and HIV prevention					
Yes*	—	—	—	0.29 (0.08–1.11)	—
CSA*: contraception use					
Yes*	—		—	—	1.08 (0.15–7.94)

SRHE, sexual and reproductive health education; CSA, childhood sexual abuse.

All models were adjusted for age, gender, residence, employment, education, mother's education, and family income.

*** *P* < 0.001; **P < 0.01; *P < 0.05.

## Discussion

Based on a nationally representative survey, this study first investigated the association between CSA experience and early sexual debut among Chinese youth. Our results showed that 21.5% of Chinese unmarried youth had made their sexual debut before 18 years old. One study from Wuhan city suggested that 10.7% of female university students had their first sexual intercourse before 18 years old (4), another study suggested that 5.9% of female university students had their sexual debut also before 18 years old (6), and another study from Ningbo city found that 43.0% of male university students had initiated sexual intercourse before college (25). The national-level data used in our study made the result more representative for the issue in the country. The prevalence of early sexual debut among Chinese youth was lower than that among those in most Western societies (7, 26), but it represents a marked increase compared with that of previous Chinese generations. This may be explained not only by the decline in average age at puberty among Chinese adolescents (27), but also by the context of moderation in societal perceptions of premarital sexual activity, as discussed earlier. This finding justifies the public concern for the potential health risks of early sexual initiation among youth in China.

Consistent with previous studies in the United States (28, 29) and Norway (30), a history of CSA was associated with an elevated risk of early onset of sexual intercourse among Chinese youth. Two separate pathways have been suggested by recent research to explain the influence of sexual abuse, and these are biological and psychological pathways. From the perspective of developmental neurobiology (31), traumatic stress may operate through the biological system, affect the timing of puberty, and place children at risk for sexual precocity (32). Also, the association between sexual abuse and early puberty has been demonstrated by one study on the sexual trajectory of neglected and abused children (13). More empirical evidence has suggested a psychological pathway. Previous research has indicated that CSA victims always have difficulty in forming healthy interpersonal relationships and are more likely to get involved in premature romantic relationships to meet their intimacy needs and heighten their feeling of belonging (33). In addition, due to the cross-sectional design of the data in our study, the association between CSA and early sexual debut can also be explained in an opposite way—youths who initiate sexual intercourse at early adolescence are at a higher risk of sexual exploitation including sexual abuse, which has also been demonstrated by one study from Sweden (34). Our finding highlights the need for making a targeted intervention of subsequent negative outcomes for both early starters of sexual intercourse and CSA victims in China.

It is also worth mentioning that more than half of sexually active youth in this survey never received any type of school-based sexual and reproductive health education, which is consistent with that of a previous study (24). For the other half, the received sexuality education mainly focused on reproductive health knowledge, and only a small proportion of youth received school-based education on STD and HIV prevention and contraception use. This result further indicates the serious lag of sexuality education and the failure to incorporate its guidelines into the curriculum for youth in China’s schools. School-based SRHE has been implemented since 1985, and it has also been reinforced as an obligation by the population and family planning law enacted by the Chinese government in 2002 (35). However, the progress on this score has been quite disappointing—sex has always been and still considered as a sensitive and taboo subject for classroom discussion, and most teachers are reluctant to provide lectures on the subject (22). A survey conducted in Shanghai reported that less than 20% of high school students received sex education from their teachers (22). In addition, knowledge about contraception use and how to reduce risky sexual behaviors are excluded from the curriculum in most Chinese schools, as it is believed that access to such information may prompt early sexual activity among adolescents (35). This finding underlines the urgent need for providing early and timely sexuality education, especially skills and information about contraception and prevention of STD and HIV for the youth.

The multivariate analysis including the interaction terms between CSA and SRHE showed that receiving any type of sexuality education—reproductive health, STD and HIV prevention, or contraception use—was not associated with a decreased risk of early initiation of sexual intercourse among youth with a history of CSA in China. Several reasons should be considered to account for this fact. First, it has been suggested that school-based sexuality education in the country is inadequate and incomprehensive due to insufficient class hours and a lack of professional teaching resources (23), which is proven by the fact that less than 30% of university students have a sound knowledge of HIV transmission (36). Second, most of the school-based education curriculums are designed only for general children and adolescents without considering CSA victims as part of them (17). Evidence shows that abused and nonabused children differ in their responses to materials related to sexual knowledge, which may act as a stimulus and cause emotional distress in abused children, which may further influence their acquisition of sexual health knowledge (37). In addition, there are studies pointing out that sex education is not an effective intervention to delay the timing of sexual debut among adolescents (23, 38). This is also supported by the results in our study that education on STD and HIV prevention and contraception use is associated with an elevated risk of early sexual debut. This finding suggests that current school-based sex education should not be considered as a substitute for the sexual education component of CSA victim treatment. Rather, the coverage of such education for them should be planned appropriately and monitored closely in the treatment setting to address their special needs and process their emotional reactions moderately so that the information would be better retained in their minds (17).

### Limitation

The present study has several limitations. First, due to the cross-sectional design of the survey, our results do not provide direct evidence of causality and therefore should be interpreted with caution. Second, the issue of sex remains a sensitive and taboo topic in Chinese society and the data were collected through a self-report; therefore, the responses to CSA experience and the age of sexual debut are subject to error of recall and social-desirability bias. Third, we are unable to obtain more detailed information about sexuality education for more specific analyses such as the age of receiving such education and the course duration, and so on. Despite these limitations, our study provides evidence for determining the association between CSA and early sexual debut among Chinese youth by taking into account the role of sexuality education using a nationally representative survey that could be applicable to other similar cultural and societal contexts.

## Conclusion

To sum up, the results of this study indicate a greater risk of early sexual debut among Chinese youth with a history of CSA and to some extent suggest that the current school-based sexuality education is inadequate to act as an effective intervention to delay sexual initiation, either for CSA victims or for the general youth. Targeted intervention with a timely and adequate course of sexuality education for both early starters of sexual intercourse and CSA victims, as well as future research evaluating such intervention programs in China, is warranted.

## Data Availability

The datasets presented in this article are not readily available because the access to these datasets lies within the purview of the Institute of Population Research, Peking University. Requests to access the datasets should be directed to the corresponding author.
